# Omidenepag, a non-prostanoid EP2 receptor agonist, induces enlargement of the 3D organoid of 3T3-L1 cells

**DOI:** 10.1038/s41598-020-72538-x

**Published:** 2020-09-29

**Authors:** Yosuke Ida, Fumihito Hikage, Araya Umetsu, Haruka Ida, Hiroshi Ohguro

**Affiliations:** Departments of Ophthalmology, Sapporo Medical University School of Medicine, Sapporo, Japan

**Keywords:** Eyelid diseases, Glaucoma

## Abstract

2D and 3D cultures of 3T3-L1 cells were employed in a study of the effects of Omidenepag (OMD), interacting with a non-prostanoid EP2 receptor, on adipogenesis. Upon adipogenesis, the effects on lipid staining, the mRNA expression of adipogenesis-related genes (*Pparγ, CEBPa*, *Ap2,* and *Glut4*) and the extracellular matrix (ECM) including collagen type 1, 4 and 6, and fibronectin, and the size and physical property of 3D organoids were compared between groups that had been treated with EP2 agonists (butaprost and OMD) and PGF2α. Upon adipogenesis, these significantly suppressed lipid staining and the mRNA expression of related genes. EP2 agonists and PGF2α influenced the mRNA expression of ECM in different manners, and these effects were also different between 2 and 3D cultures. Examining the physical properties by a microsqueezer indicated that the solidity of the 3D organoids became significantly lowered upon adipogenesis and these effects were not affected by EP2 agonists. In contrast, 3D organoid stiffness was markedly enhanced by the presence of PGF2α. These observations indicate that EP2 agonists affect the adipogenesis of 3T3-L1 cells in different manners, as compared to PGF2α, suggesting that OMD may not induce PGF2α related orbital fat atrophy, called the deepening of the upper eyelid sulcus (DUES).

## Introduction

It is well known that prostaglandins (PGs) are involved in various physiological and pathological functions such as regulation of smooth muscles, inflammation, immune response, and others^1^. Camras et al. first reported that the topical administration of PGF2α and PGE2 decreases intraocular pressure (IOP) in rabbits, and suggested that the stimulation of the prostanoid FP receptors and PGE2 receptors (mainly EP2 and EP3) represent a potentially novel therapy for the treatment of patients with ocular hypertensive (OH) and glaucoma^2^. As of this writing, several PGs that affect prostanoid FP receptors have developed and are recognized as first-line medications because of their few systemic side effects in addition to their efficacy for decreasing elevated IOPs^3–5^. It has recently been recognized that the longterm use of FP agonists causes prostaglandin-associated periorbitopathy (PAP) including deepening of the upper eyelid sulcus (DUES), elongation of eyelashes, hyperpigmentation of the skin, and changes in iris color, in a considerable number of patients^6–9^. Among these, DUES is cosmetically the most annoying side effect. A magnetic resonance imaging study reported that a significant reduction in orbital adipose tissues may result in DUES^10^.

Omidenepag isopropyl (OMDI), a prodrug, is hydrolyzed in the eye to the active form (Omidenepag, OMD) which functions as a selective, nonprostaglandin, prostanoid EP2 agonist, was recently developed and is now being used in the treatment of patients with OH and glaucoma^11,12^. Studies using OH monkeys demonstrated that the dynamics of the pharmacokinetics of OMD in the aqueous humor (AH) are quite different from those of FP agonists although they are categorized as members of the prostanoid receptor family of agonists^11^. Therefore, based upon these facts, it would be of great interest to examine the issue of whether OMD may cause DUSE or not. Yamamoto et al. recently examined the effects of OMD on adipogenesis in 3T3-L1 cells in conventional 2D cultures, and found that OMD had no effect on adipocyte differentiation^12^.

In human organs, since adipocytes are spread three-dimensionally (3D) in space, a 3D cell culture system would be expected to provide more relevant information than a conventional two-dimension (2D) cell culture^13–15^. In fact, quite recently, such 3D tissue cultures have been used in conjunction with several human disease models^14^. Using the 3D organoid culture technique, in our previous studies, we found that PGF2α significantly affected adipogenesis and the extracellular matrixes (ECMs) in 3T3-L1 cells^16^ as well as human orbital fibrobrasts (HOFs)^17^. Therefore, in the current study, using a replicated pathogenic DUES model by 2D and 3D cultures using 3T3-L1 cells, we analyzed and compared the effects of FP and EP agonists, OMD and butaprost (Buta), on adipogenesis, ECM expression, and the sizes and physical properties of the 3D organoids.

## Results

**Figure 1:**
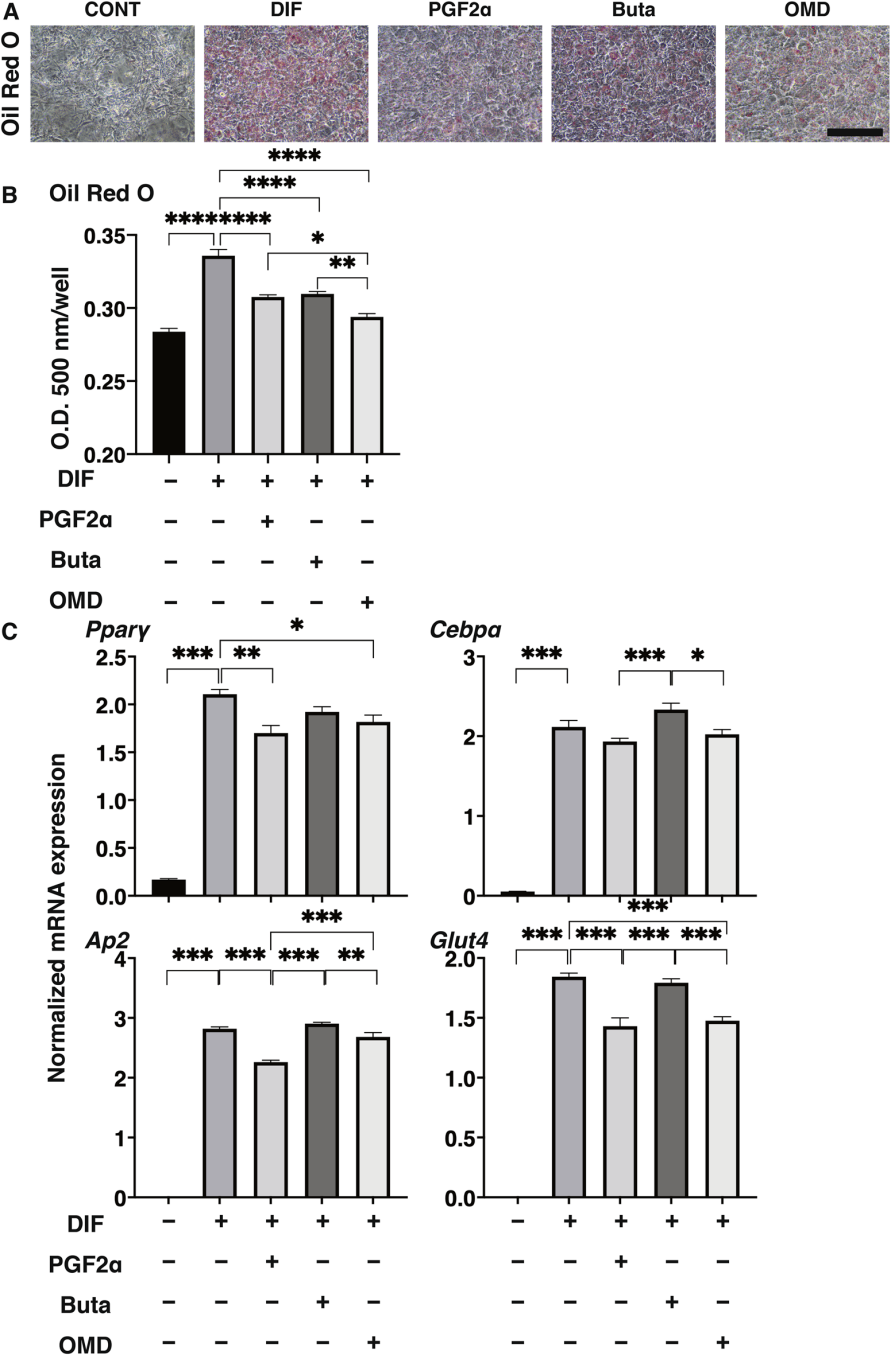
Effects of PGF2α, butaprost (Buta) or omidenepag (OMD) on adipogenesis of 2D cultured 3T3-L1 cells.

**Figure 2:**
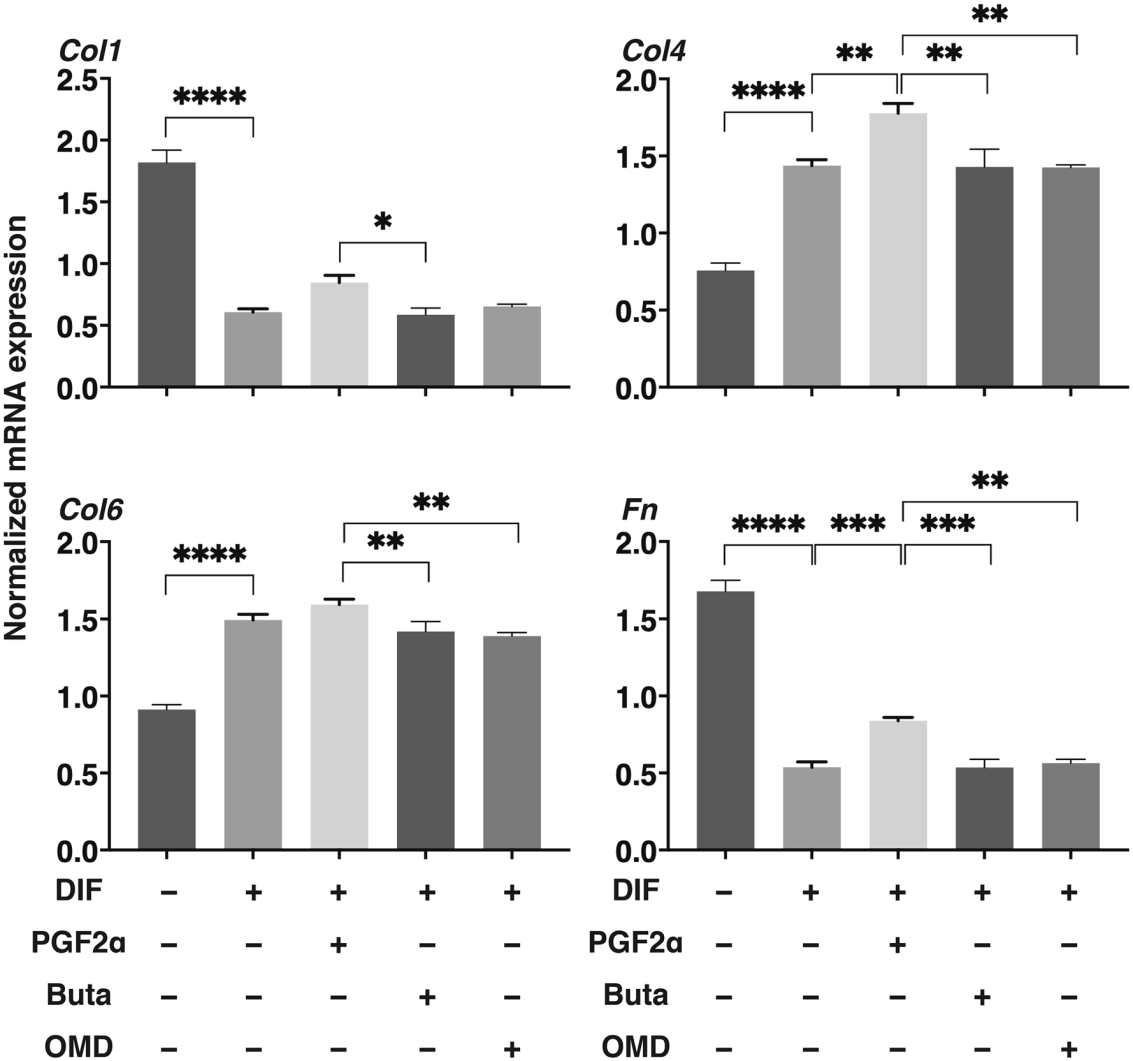
Effects of PGF2α, butaprost (Buta) or omidenepag (OMD) on ECM expression in 2D cultured 3T3-L1 cells.

To compare pharmacological effects between PGF2α and EP receptor agonists toward adipogenesis of the 2D cultured 3T3-L1 cells, lipid staining by Oil Red O, and the mRNA expression of adipogenesis related genes including *Pparγ*, *Cebpα, Ap2* and *Glut4*, and major ECM (*Col 1*: collagen 1, *Col 4*: collagen 4, *Col 6*: collagen 6, *Fn*: fibronectin) were evaluated in the presence of PGF2α or EP receptor agonists (Buta: butaprost, OMD: Omidenepag). As shown in Fig. 1, the staining of 2D cultured 3T3-L1 cells by Oil Red O and the gene expression of all adipogenesis related genes tested were significantly enhanced upon adipogenic differentiation (DIF), and these effects were markedly or slightly inhibited by the presence of PGF2α or OMD, respectively. In terms of the mRNA expression of ECM, significant suppression and enhancement was observed in *Col 1* and *FN*, and *Col 4* and *Col 6*, respectively upon DIF (Fig. 2). In the presence of PGF2α, *Col4* and *Fn*, and *Col1* were significantly or relatively enhanced as compared to DIF. While, in contrast, Buta and OMD did not cause any changes (Fig. 2). These data indicate that EP agonists caused similar effects toward lipid metabolism in the 2D cultured 3T3-L1 cells, but were different toward their ECM expressions as compared to PGF2α.

**Figure 3:**
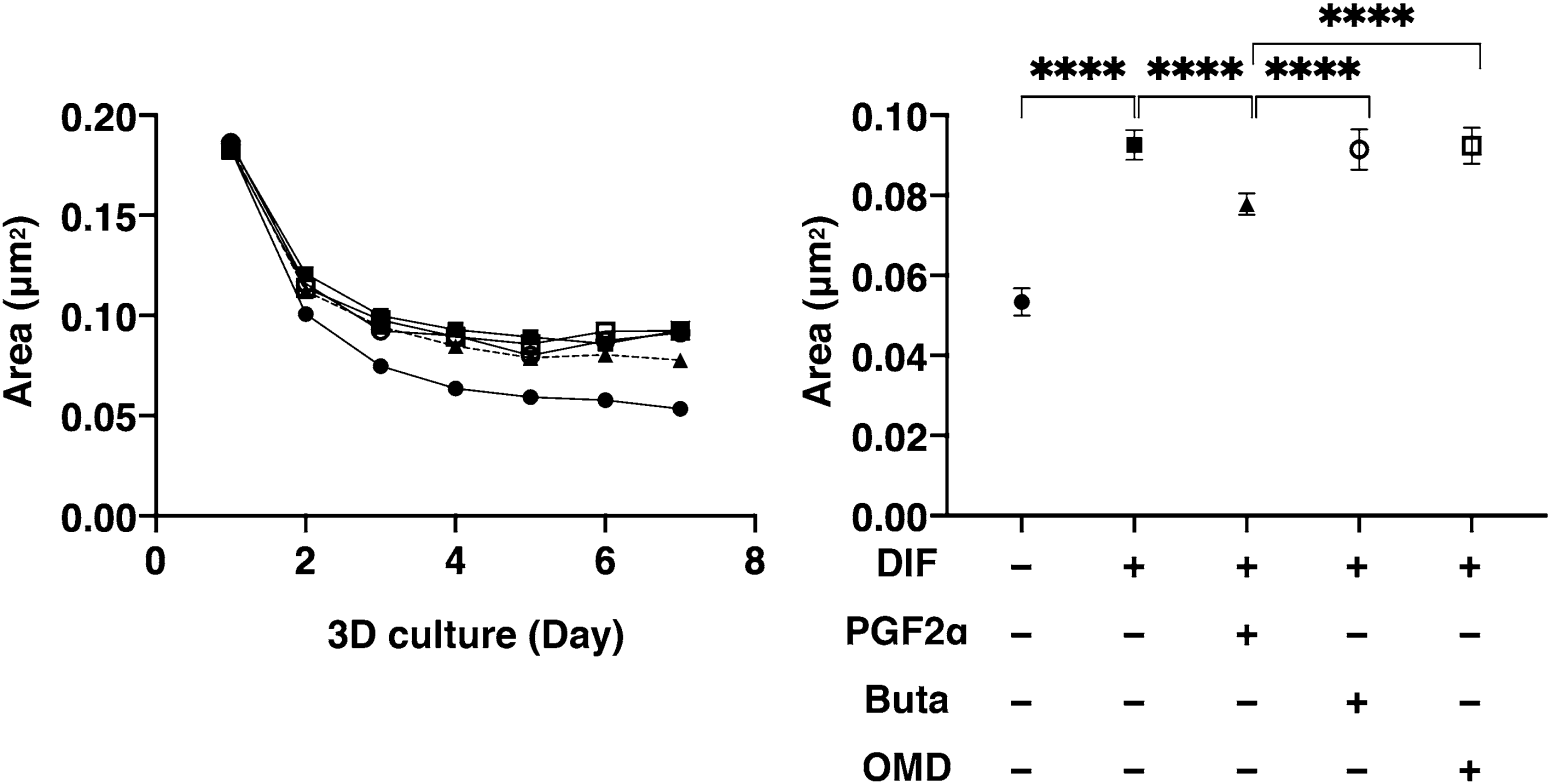
Effects of PGF2α, butaprost (Buta) or omidenepag (OMD) on sizes of 3T3-L1 3D organoid during their adipogenesis.

**Figure 4:**
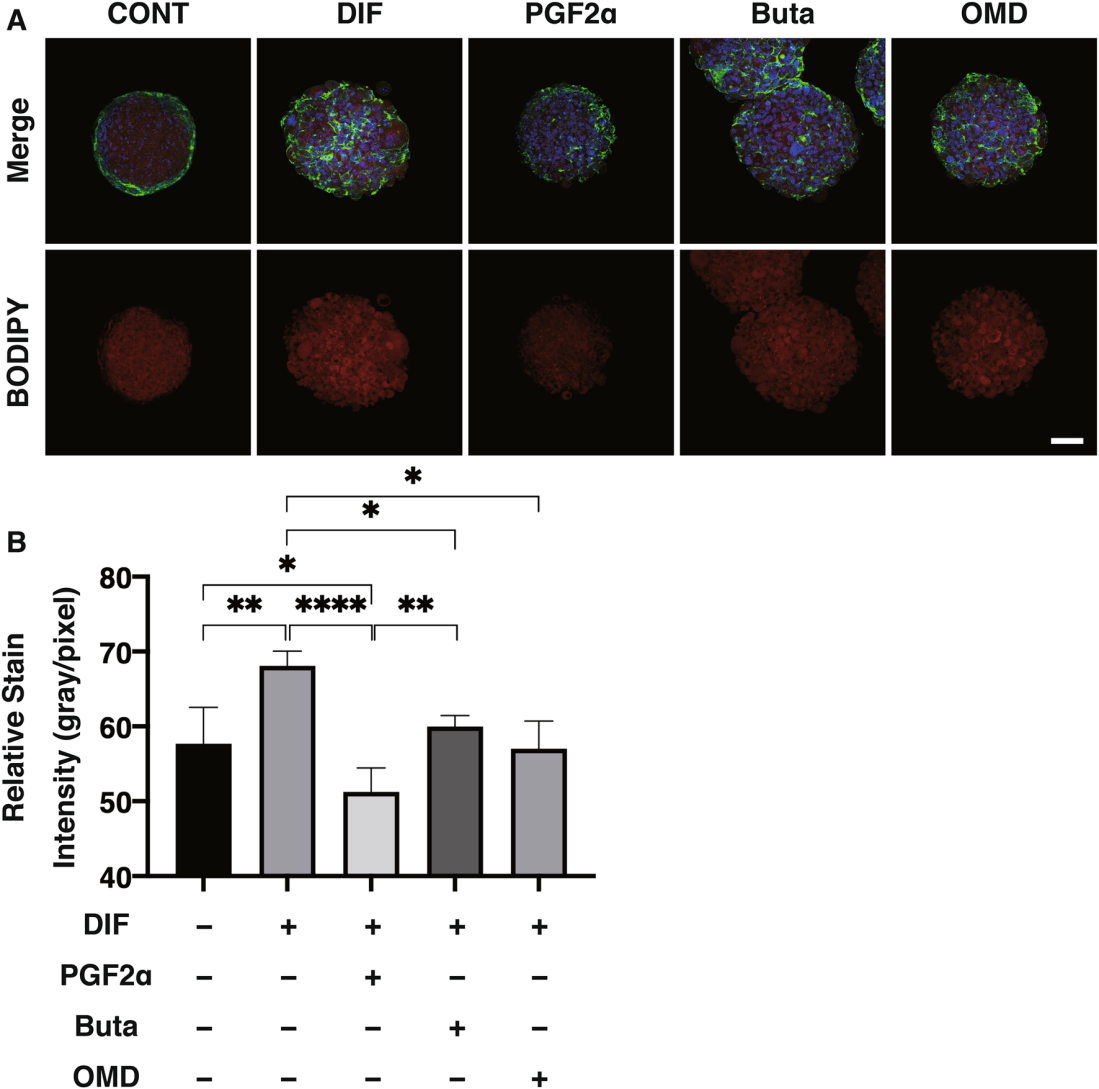
Representative confocal images of lipid staining (BODIPY) of the 3D organoids under various conditions.

**Figure 5:**
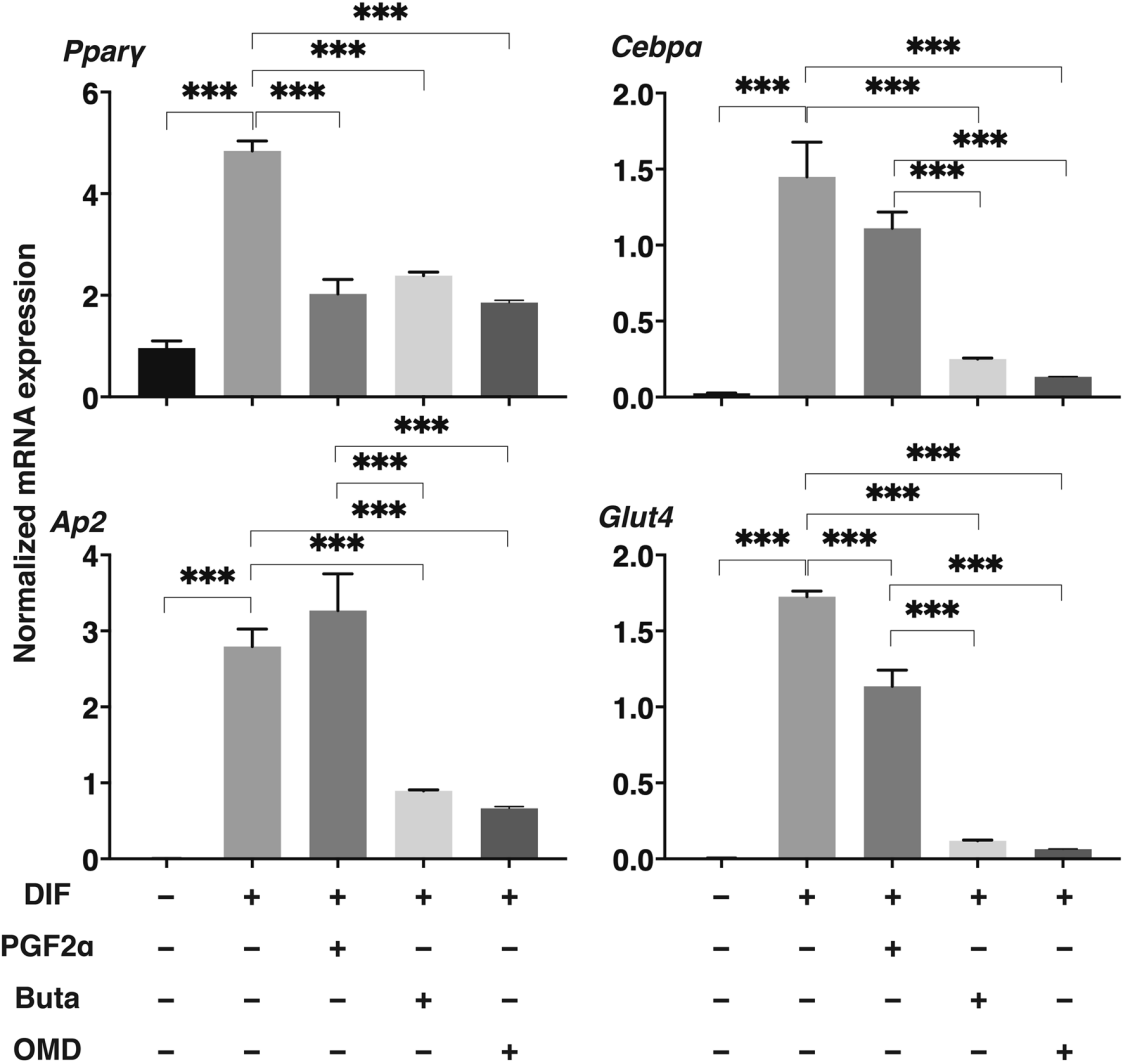
mRNA expression of adipogenesis related genes including *Pparγ, Cebpα, Ap2 or Glut4,* of the 3D organoids under various conditions.

To study this issue further, 3D organoid cultures of 3T3-L1 cells which are known to be a more representative disease model for DUES^16^, were used. Consistent with our preceding study, the sizes of the uniform round-shape spheroidal organoids of 3T3-L1 cells were smaller than the matured forms during the 7-day culture (Fig. 3). The mean sizes were significantly larger upon DIF. These effects by DIF were greatly suppressed by PGF2α but not by Buta nor OMD. Lipid staining intensity by BODIPY (Fig. 4) were significantly enhanced upon DIF, but such DIF induced changes were markedly suppressed by the presence of either PGF2α, Buta or OMD. As shown in Fig. 5, the mRNA expressions of *Pparγ*, *Cebpα, Ap2* and *Glut4* were also significantly increased by DIF. Those DIF induced changes of all four genes or all for genes except *Ap2* were marked suppressed by EP2 agonists or PGF2α, respectively. These effects of PGF2α and EP agonists toward lipid metabolism in 3D organoids were more evident in the lipid staining by BODIPY and the mRNA expressions of *Pparγ*, as that observed in the 2D cultured experiment, as above, were found, although some differences were observed in the mRNA expressions of *Cebpα, Ap2* and *Glut4* between 2 and 3D culture experiments.

**Figure 6:**
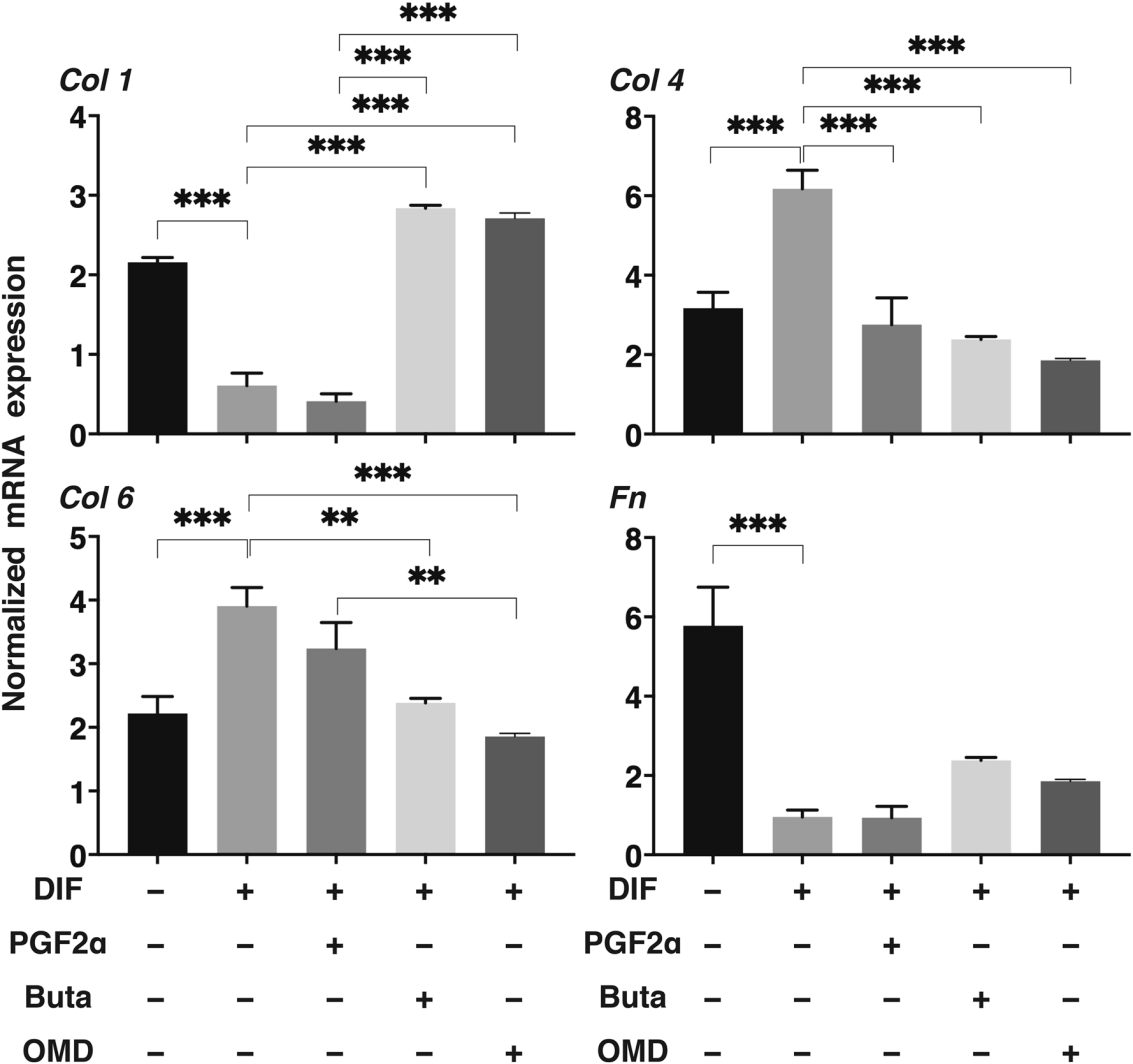
qPCR analyses of the gene expression of ECM in 3D organoids.

To elucidate effects of PGF2α and EP agonists on the gene expression of the ECM during the adipogenesis of the 3D organoids, those of the major ECM as above were examined (Fig. 6). A significant suppression or enhancement was observed in those of *Col 1* and *Fn*, or *Col 4* and *Col 6*, respectively, upon DIF. Those changes were similar to the values obtained in the 2D culture experiments as above. In contrast, the effects of PG and EP agonists on 3D organoids were markedly different than the values for the 2D culture experiments. That is, in the presence of PGF2α, changes in the mRNA expression of ECM were as follows; *Col 1* (2D: enhancement, 3D: suppression), *Col 4* (2D: marked enhancement, 3D: marked suppression), *Col 6* (2D: enhancement, 3D: suppression) and *Fn* (2D: marked enhancement, 3D: no effect), respectively as compared to those in DIF. In the presence of Buta or OMD, although the values for ECM were not altered in the 2D culture experiment as above, a significant enhancement was observed in the case of *Col 1* and *Fn*, and a relative or significant suppression was found in *Col 4* and *Col 6*, respectively.

**Figure 7:**
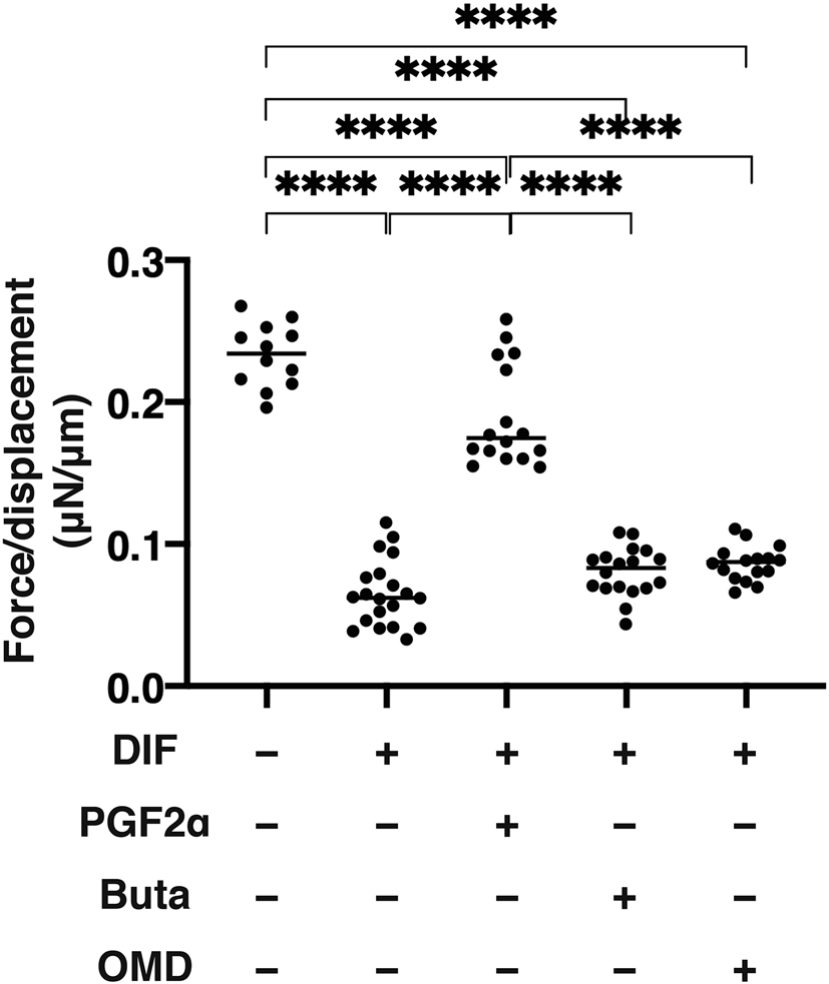
Physical solidity of 3D organoids.

To study physical solidity of the 3D organoids under several conditions, the force required (μN) to cause the 3D organoid size to form a semi-diameter was measured upon mechanical compression by a microsqueezer analysis. As shown in Fig. 7, much lower forces were required upon DIF as compared with those of preadipocytes. PGF2α induced a significant stiffness of the organoid whereas Buta or OMD had no effect on their solidity.

2D cultures of 3T3-L1 cells were performed under several conditions; preadipocytes of the 3T3-L1 cells (DIF-) and their adipogenic differentiation (DIF +) without or with 100 nM of PGF2α (PGF2α), 100 nM butaprost (Buta) or 100 nM Omidenepag (OMD). These cells were stained with Oil Red O (panel A, scale bar: 100 μm) and their staining intensities (O.D. at 500 nm/well) were plotted (panel B). The mRNA expression　of adipogenesis related genes including *Pparγ*, *Cebpα, Ap2* and *Glut4* of above conditions were plotted in panel C. All experiments were performed in triplicate using fresh preparations. Data are presented the arithmetic mean ± standard error of the mean (SEM). *P < 0.05, ** P < 0.01, ***P < 0.005, ****P < 0.001 (ANOVA followed by a Tukey’s multiple comparison test).

2D cultured 3T3-L1 preadipocytes (DIF-) and their adipogenic differentiation (DIF +) without or with 100 nM PGF2α (PGF2α), 100 nM butaprost (Buta), or 100 nM Omidenepag (OMD) were subjected to qPCR analysis to estimate mRNA expression of ECM (*Col 1*: collagen 1, *Col 4*: collagen 4, *Col 6*: collagen 6, *Fn*: fibronectin). All experiments were performed in triplicate using fresh preparations. Data are presented as the arithmetic mean ± standard error of the mean (SEM). *P < 0.05, **P < 0.01, ***P < 0.005, ****P < 0.001 (ANOVA followed by a Tukey’s multiple comparison test).

The mean sizes of 3D organoids of 3T3-L1 preadipocytes (DIF-, closed circles) and their adipogenic differentiation (DIF + , closed squares) without or with 100 nM PGF2α (PGF2α, closed triangles), 100 nM butaprost (Buta, open circles) or 100 nM Omidenepag (OMD, open squares) were measured. Fluctuations of the samples during a 7-day culture plotted (left panel) and those at Day 7 were compared among the experimental groups (right panel). All experiments were performed in triplicate using fresh preparations consisting of 16 organoids each. Data are presented as arithmetic means ± standard error of the mean (SEM). ****P < 0.001 (ANOVA followed by a Tukey’s multiple comparison test).

3D organoids at Day 7 under various conditions; preadipocytes of the 3T3-L1 cells (DIF-) and their adipogenic differentiation (DIF +) without or with 100 nM of PGF2α (PGF2α), 100 nM butaprost (Buta) or 100 nM Omidenepag (OMD) were immunostained by BODIPY (red), DAPI (blue) and phalloidin (green) (A), and their staining intensities by BODIPY were plotted (B). All experiments were performed in duplicate using fresh preparations consisting of 10 organoids each. Data are presented as the arithmetic mean ± standard error of the mean (SEM). *P < 0.05, **P < 0.01, ****P < 0.001 (ANOVA followed by a Tukey’s multiple comparison test).

At Day 7, 3D cultures organoids of 3T3-L1 preadipocytes (DIF-) and their adipogenic differentiation (DIF +) without or with 100 nM Prostaglandin F2α (PGF2α), 100 nM butaprost (Buta) or 100 nM Omidenepag (OMD) were subjected to qPCR analysis to estimate mRNA expression of adipogenesis related genes including *Pparγ*, *Cebpα*, *Ap2* and *Glut4*. All experiments were performed in duplicate using fresh preparations consisted of 16 organoids each. Data are presented as the arithmetic mean ± standard error of the mean (SEM). ***P < 0.005 (ANOVA followed by a Tukey’s multiple comparison test).

At Day 7, 3D culture organoids of 3T3-L1 preadipocytes (DIF-) and their adipogenic differentiation (DIF +) without or with 100 nM Prostaglandin F2α (PGF2α), 100 nM butaprost (Buta) or 100 nM Omidenepag (OMD) were subjected to qPCR analysis to estimate mRNA expression of ECM (*Col 1*: collagen 1, *Col 4*: collagen 4, *Col 6*: collagen 6, *Fn*: fibronectin). All experiments were performed in duplicate using fresh preparations consisting of 16 organoids each. Data are presented as arithmetic means ± standard error of the mean (SEM). **P < 0.01, ***P < 0.005 (ANOVA followed by a Tukey’s multiple comparison test).

Among the experimental groups under several conditions; preadipocytes of the 3T3-L1 cells (DIF-) and their adipogenic differentiation (DIF +) without or with 100 nM of PGF2α (PGF2α), 100 nM butaprost (Buta) or 100 nM Omidenepag (OMD), physical solidity of their 3D organoids at Day 7 are analyzed by means of a microsqueezer (μN/μm force/displacement). All experiments were performed in duplicate using fresh preparations consisted of 12–19 organoids. Data are presented as arithmetic means ± standard error of the mean (SEM). ***P < 0.005 (ANOVA followed by a Tukey’s multiple comparison test).

At Day 7, 2D or 3D cultures 3T3-L1 cells with adipogenesis (DIF +) were subjected to qPCR analysis to estimate mRNA expression of FP (*Ptgfr*) or EP (*Ptger2*) receptor. All experiments were performed in duplicate using fresh preparations. Data are presented as the arithmetic mean ± standard error of the mean (SEM). ***P < 0.005 (ANOVA followed by a Tukey’s multiple comparison test).

## Discussion

PGE2 synthesized by cyclooxygenases and the cytosolic and microsomal PGE synthases linked to four G protein-coupled receptor subtypes, designated as prostaglandin receptors EP 1–4^18^. Functionally EP1 induces the influx of calcium and enhances intracellular free calcium^19^. EP2 and EP4 predominantly mediates increases in cAMP levels, whereas EP3 inhibits cAMP production. In the human, each EP receptor subtype is distributed to different organelles; EP1: myometrium, pulmonary veins, colon, skin, mast cells; EP2: leukocytes, smooth muscle, central nervous system (CNS), reproductive system, bones; EP3: CNS, cardiovascular system, reproductive system, kidney, urinary bladder; EP4: leukocytes, smooth muscle, cardiovascular system and bones^20^. In addition to this, these EP species also exert their action via different regulatory mechanisms and signal transduction pathways. Among these, the EP2 receptor has been shown to be involved in IOP regulation in several in vivo studies and EP2 receptor agonists cause ocular hypotensive effects on elevated IOPs^11^. In the present study, we also found positive expressions of EP2 receptor (*Ptger2*) within 2D and 3D cultured 3T3-L1 cells (Supplemental Fig. 1), and such expression was significantly enhanced by the adipogenesis. Taprenepag isopropyl (TPI), a potent and selective EP2 receptor agonist^21^ was evaluated in a Phase II controlled randomized trial, by topically applying them to patients with glaucoma or OH^22^. The TPI monotherapy significantly reduced their IOPs to levels comparable to LAT. In addition, TPI displayed additive effects to LAT, suggesting their possible use in combination therapy in the treatment of glaucoma and OH. OMDI, a non‐prostanoid isopropyl ester derivative is hydrolyzed to OMD during its corneal penetration with little or no activity on EP4 receptors^23^. OMDI was reported to have strong effects in lowering IOP in various animal models of OH and glaucoma^11^. In a randomized, investigator‐masked, active‐controlled multi-center phase III trial in Japan, the lowering effects of IOP for OMDI were comparable to those by LAT, although the IOP fluctuation from the baseline in the mean diurnal IOP for OMDI was 0.63 mmHg lower than that for LAT^24^. These findings suggest that a selective EP2 agonist OMDI may potentially be similarly beneficial as that for an anti-glaucoma medication as a FP2 agonist. In the current study, we investigated the issue of whether OMDI causes DUSE or not similar to PGF2α, and found following results; 1) the lipid staining and mRNA expression of genes related to adipogenesis were significantly enhanced upon DIF and this was marked suppressed by the presence of PGF2α, Buta or OMD in both 2D and 3D culture of 3T3-L1 cells, 2) the mean sizes of 3D organoids of 3T3-L1 cells were significantly larger upon DIF, and these effects by DIF were greatly suppressed by PGF2α but not by Buta or OMD, 3) a significant enhancement or suppression was observed in the mRNA expression of *Col 1* and *Fn*, or *Col 4* and *Col 6*, respectively, upon DIF in both 2D and 3D, 4) in the presence of PGF2α, mRNA expressions of all four ECM were enhanced, but not altered by Buta or OMD as compared to DIF in the 2D culture experiment, 5) although, in 3D organoids, the mRNA expression of all four ECM were suppressed in the presence of PGF2α, further enhancement in *Col 1* and *Fn*, and suppression in *Col 4* and *Col 6* were observed in the presence of Buta or OMD as compared to DIF, 6) the stiffness of the organoids was markedly softened upon DIF, and such DIF-induced effects were significantly inhibited by PGF2α, but were not affected by Buta or OMD.

Yamamoto et al. independently investigated the effects of OMD on the adipogenesis of 2D cultured 3T3-L1 cells by comparing with the effects of PGF2α and reported the following findings; (1) the lipid staining by Oil Red O was significantly inhibited by LAT and PGF2α, but not by OMD, and (2) LAT and PGF2α significantly suppressed adipogenesis related gene expression, but OMDI had no effect^12^. In terms of the effects of PGF2α on adipogenesis, our present results are in good agreement with findings reported by Yamamoto et al. in addition to a number of previous studies^25,26^. In contrast, however, the effects of OMD on adipogenesis of the 3T3-L1 cells differed between the Yamamoto et al. study^12^ using 2D culture (no effect) and our present study in which both 2D and 3D cultures were used (suppression). We speculate that the difference in these results can likely be attributed to differences in the methodology being used. In the Yamamoto et al. study^12^, the rates of Oil Red O positive staining areas, calculated by using microscopy, were compared among experimental groups without using a positive control for the EP2 agonist (Buta). In our current study, a more accurate method was used, i.e., by measuring the Oil O Red dye concentrations in addition to the use of Buta. Furthermore, consistent observations of EF2 agonists (OMD and Buta) toward lipid staining levels and the mRNA expression of adipogenesis related genes (*PPrγ* , *Cebpα*, *Ap2* and *Glut4*) were obtained in both 2D and 3D culture experiments. In fact, a number of studies have demonstrated that PGE2 and PGF2α, both inhibit adipocyte development^27,28^. However, the suppression efficacy of the EP2 agonists (OMD and Buta) was significant, but less than that of PGF2α in both 2D and 3D cultured 3T3-L1 cells as shown in Figs. 1, 4 and 5. Since it is well known that stimulation of the EP2 receptor leading Gs mediated elevation of cAMP concentrations induced stimulation of the adipogenesis^20^, this mechanism may be involved in the difference in the efficacy between EP2 agonists and PGF2α toward adipogenesis as above.

Although the molecular mechanisms responsible for causing difference in the pharmacological effects toward ECM expressions of the 3T3-L1 cells between PGF2α and EP 2 agonists have not been elucidated, the findings reported herein reveal that both agonists regulated intracellular function in different manners. ECM essentially provides structural support as well as a number of cellular functions that are critically regulated by several factors, such as matrix metalloproteinase (MMP), tissue inhibitors of metalloprotease (TIMP), PGs, and others^29,30^. As major ECM, collagens (COLs) comprised of triple helical structures and COL-related proteins are present in the ECM and at the interface between a cell and ECM^31^. COL 1 is the most abundant protein in vertebrates. COL 4 is the basement membrane-rich ECM^32^. COL 6 has several distinct cellular functions such as providing biomechanical regulatory signals in cell survival processes and in the differentiation of several types of cells^33^. FN is secreted as a dimer, and contains a self-assembly domain that induces the formation of a three-dimensional (3D) matrix called “fibrillogenesis”^31^. Pathologically, FN fibrillogenesis may be involved in several fibrotic diseases^31^. These COL 1, 4, and 6, and FN are all present and play pivotal roles during adipogenesis in vivo and in vitro. In fact, our previous study using 3D organoids from 3T3-L1^16^ and HOF^17^ demonstrated the following: (1) the down-regulation of COL 1 and FN, and the up-regulation of COL 4 and COL6 expression following differentiation (DIF), and (2) the down-regulation of all four ECM by the presence of PGF2α as compared to DIF. These observations were again confirmed in the present study. In the presence of EP2 agonists, a significant upregulation of COL 1 and FN were observed, and these changes were markedly different from those for PGF2α. In contrast, EP2 agonists, OMD and Buta induced quite different changes in these ECM expressions, as described above. Furthermore, these changes in ECM expression also were different between the 2D and 3D culture experiments. This observation was not surprising because in our precedent study using trypsin digestion^16^, the breakdown times of 3D organoids from preadipocytes of 3T3-L1 cells were much longer than the corresponding values for a similar 2D cell culture, and the values for 3D organoids were further enhanced upon adipogenesis. Furthermore, we also performing quite unique analyses, i.e. measuring the physical stiffness of the 3D organoids by means of a microsqueeser, substantial differences in physical properties were found between the 3D organoids treated with PGF2α and EP2 agonists. This is the only currently available method that permits the stiffness of the 3D organoids to be measured because this permits data to be collected on a single living 3D organoid.

Taken together, although long-term evidence in patients is needed, the current data suggests that OMD does not induce DUES in glaucoma and in OH patients unlike FP agonists. Adverse effects of OMDI include a potential risk for cystoid macular edema was advocated especially in patients who had undergone cataract surgery with intraocular lens implantation or who are aphakic^34,35^. These suggests that additional currently unknown mechanisms appear to be involved. In addition, the physiological properties of human orbital fatty tissues may be different from those of 3T3-L1 cells. Therefore, further studies involving characterizing the pharmacological and pathological aspects of OMD and other EP2 agonists using other sources of samples including human orbital fibroblast (HOF) will be our next project.

## Materials and methods

### Chemicals and drugs

High Glucose Dulbecco’s Modified Eagle’s Medium (HG-DMEM) (# 11965092, Gibco/Thermo Fisher Scientific, Waltham, MA), fetal bovine serum (FBS) (# 16-000-044, Gibco/Thermo Fisher Scientific), calf serum (CS)(#S0400, Biowest), L-glutamin (# 25030081, Gibco/Thermo Fisher Scientific), antibiotic/antimycotic (# 15240062, Gibco/Thermo Fisher Scientific), penicillin/streptomycin (# 15140122, Gibco/Thermo Fisher Scientific), Ficoll-Paque Plus (# 17-1440-03, GE Healthcare, Piscataway, NJ), Puromycin (# P8833, Sigma-Aldrich, St Louis, MO), Protamine sulfate salt from salmon (# P4020, Sigma-Aldrich), Methocel A4M (# 94378, Sigma-Aldrich), Dexamethasone (# D1756, Sigma-Aldrich), 3,3′,5-Triiodo-L-thyronine (T3) (# T6397, Sigma-Aldrich), Troglitazone (# 71750, Cayman Chemical, Ann Arbor, MI), Porcine insulin (# I5523, Sigma-Aldrich), Prostaglandin F2α (#16010, funakoshi, Tokyo, Japan), omidenepag (generous gift from Santen Pharmaceutical Co., Ltd., Osaka, Japan), butaprost free acid (#13741, funakoshi, Tokyo, Japan).

### Two-dimension (2D) and three-dimension (3D) culture of 3T3-L1 cells and their adipogenic differentiation in the presence or absence of prostaglandin F2α (PGF2α), omidenepag (OMD) or butaprost (Buta)

The 3T3-L1 preadipocytes (#EC86052701-G0, KAK) were grown in 2D culture medium (HG-DMEM containing 100 U/mL penicillin, 100 μg/mL streptomycin and 10% CS) in 150 mm dish until confluence at 37℃.

To obtain 3T3-L1 organoids, 3T3-L1 preadipocytes were grown in 3D pre-culture medium (HG-DMEM containing 8 mg/L d-biotin, 4 mg/L calcium pantothenate, 100 U/mL penicillin, 100 μg/mL streptomycin and 10% CS) in 150 mm dish as above. When the cultures reached approximately a 90% confluence, the cells were washed with phosphate buffered saline (PBS), detached using 0.25% Trypsin/EDTA and resuspended in 3D pre-culture medium containing 0.25% w/v Methocel A4M (3D organoid medium). Approximately 20,000 cells in the 28μL 3D organoid medium were placed into each well of the drop culture plate (defined as 3D/Day 0). On alternate days, 14 μL of the medium was substituted with 14 μL of fresh medium in each well.

For the induction of adipogenic differentiation, two days after the 2D cells in the 2D culture medium or 3D organoids at Day 1 in the 3D organoid medium reached confluence, there were processed by supplementation with 250 nM dexamethasone, 10 nM T3, 10 μM troglitazone, and 1 μg/ml insulin during the initial two days, and during the following 4 days with 10 μM troglitazone and 1 μg/ml insulin. To study the efficacy of several drugs, 100 nM prostaglandin F2α (PGF2α), 100 nM omidenepag (OMD), or 100 nM butaprost (Buta) were added during their adipogenic differentiation. In terms of their corresponding receptor, PF and EP2 receptors, of these drugs, we confirmed their positive expressions with the 2D and 3D cultured 3T3-L1 cells (Supplemental Fig. 1).

Phase contrast images of the 3D organoids were captured in a × 4 objective lens using an inverted microscope (Nikon ECLIPSE TS2; Tokyo, Japan). For measurement of each organoid size, the largest cross-sectional area (CSA) was calculated using the Image-J software version 1.51n (National Institutes of Health, Bethesda, MD).

### Lipid staining by Oil Red O (2D) and BODIPY (3D) of 3T3-L1 cells

2D cultured 3T3-L1 cells, as described above, were washed with PBS and then stained with Oil Red O stain according to the protocol of a commercial kit (Abcam, #133102). Briefly, after washing with PBS, the cells were fixed in a formalin solution for 15 min, and then stained with an Oil Red O solution for 30 min at room temperature (RT). Microscope images were taken to visualize the red oil stained droplets. For quantitative analysis, the dye was extracted in isopropanol and the O.D. at 500 nm measured.

To analyze lipid droplet formation in the 3D organoids, the organoids were transferred to 6 super-low attachment well dishes, incubated in 0.2% 1:1000 dilutions of BODIPY, DAPI and phalloidin in PBS for 1 h, and then fixed in 4% paraformaldehyde (PFA) in PBS for 10 min at RT. Fluorescence intensity of the BODIPY-stained lipid droplets was measured using a Nikon A1 confocal microscope (Tokyo, Japan) and quantified using Image J software version 2.0.0 (NIH, Bethesda, MD).

### Quantitative PCR

Total RNA was extracted from 2D cultured cells within a single well out of 12 wells of the culture dish or 16 organoids using a RNeasy mini kit (Qiagen, Valencia, CA). Reverse transcription was performed with the SuperScript IV kit (Invitrogen) as per the manufacturer’s instructions. Respective gene expression was quantified by real-time PCR with the Universal Taqman Master mix using a StepOnePlus instrument (Applied Biosystems/Thermo Fisher Scientific). The quantities of cDNA were normalized to the expression of housekeeping gene 36B4 (*Rplp0*) and are shown as fold-change relative to the control. Sequences of primers and Taqman probes used are shown in Supplementary Table 1.

### Microindentation force measurement of the 3D organoids

The microindentation force of 3D organoids was measured using a microsqeeser (MicroSquisher, CellScale, Waterloo, ON, Canada) equipped with a microscale compression system composed by a 406-μm diameter cantilever, as recently reported (22). Single 3D organoids obtained under several conditions were individually placed on a 3-mm × 3-mm plate and compressed to 50% deformation during a period of 20 s. These processes were monitored by a microscopic camera as shown in Supplementary Movie 1. The force needed to achieve 50% strain was measured through a cantilever, and the data are expressed as force/displacement (μN/μm).

### Statistical analysis

All statistical analyses were performed using Graph Pad Prism 8 (GraphPad Software, San Diego, CA). For comparison of two mean values, a two-tailed Student’s t-test was used to calculate statistical significance with a confidence level greater than 95%. To analyze the difference in groups, a grouped analysis with a two-way analysis of variance (ANOVA) followed by a Tukey’s multiple comparison test was performed. Data are presented as the arithmetic mean ± standard error of the mean (SEM).

## Supplementary information

Supplementary Information.

Supplementary Movie.

## References

[1] Impagnatiello F (2019). Prostaglandin analogues and nitric oxide contribution in the treatment of ocular hypertension and glaucoma. Br. J. Pharmacol..

[2] Camras CB, Bito LZ, Eakins KE (1977). Reduction of intraocular pressure by prostaglandins applied topically to the eyes of conscious rabbits. Invest. Ophthalmol. Vis. Sci..

[3] Cheema A, Chang RT, Shrivastava A, Singh K (2016). Update on the medical treatment of primary open-angle glaucoma. Asia Pac. J. Ophthalmol. (Phila).

[4] Schwenn O, Heckmann B, Guzy C, Miller PJ (2010). Long-term effect of latanoprost/timolol fixed combination in patients with glaucoma or ocular hypertension: a prospective, observational, noninterventional study. BMC Ophthalmol..

[5] Susanna R, Giampani J, Borges AS, Vessani RM, Jordao ML (2001). A double-masked, randomized clinical trial comparing latanoprost with unoprostone in patients with open-angle glaucoma or ocular hypertension. Ophthalmology.

[6] Filippopoulos T (2008). Periorbital changes associated with topical bimatoprost. Ophthalmic Plast. Reconstr. Surg..

[7] Park J, Cho HK, Moon JI (2011). Changes to upper eyelid orbital fat from use of topical bimatoprost, travoprost, and latanoprost. Jpn. J. Ophthalmol..

[8] Tappeiner C, Perren B, Iliev ME, Frueh BE, Goldblum D (2008). Orbital fat atrophy in glaucoma patients treated with topical bimatoprost–can bimatoprost cause enophthalmos?. Klin. Monbl. Augenheilkd.

[9] Inoue K, Shiokawa M, Wakakura M, Tomita G (2013). Deepening of the upper eyelid sulcus caused by 5 types of prostaglandin analogs. J. Glaucoma.

[10] Jayaprakasam A, Ghazi-Nouri S (2010). Periorbital fat atrophy - an unfamiliar side effect of prostaglandin analogues. Orbit.

[11] Fuwa M (2018). Effects of a novel selective EP2 receptor agonist, omidenepag isopropyl, on aqueous humor dynamics in laser-induced ocular hypertensive monkeys. J. Ocul. Pharmacol. Ther..

[12] Yamamoto Y (2020). Effects of the selective EP2 receptor agonist omidenepag on adipocyte differentiation in 3T3-L1 cells. J. Ocul. Pharmacol. Ther..

[13] Chun TH (2012). Peri-adipocyte ECM remodeling in obesity and adipose tissue fibrosis. Adipocyte.

[14] Huh D, Hamilton GA, Ingber DE (2011). From 3D cell culture to organs-on-chips. Trends Cell Biol..

[15] Hikage F, Atkins S, Kahana A, Smith TJ, Chun TH (2019). HIF2A-LOX pathway promotes fibrotic tissue remodeling in thyroid-associated orbitopathy. Endocrinology.

[16] Ida Y, Hikage F, Itoh K, Ida H, Ohguro H (2020). Prostaglandin F2alpha agonist-induced suppression of 3T3-L1 cell adipogenesis affects spatial formation of extra-cellular matrix. Sci. Rep..

[17] Itoh K, Hikage F, Ida Y, Ohguro H (2020). Prostaglandin F2alpha agonists negatively modulate the size of 3D organoids from primary human orbital fibroblasts. Invest. Ophthalmol. Vis. Sci..

[18] Negishi M, Sugimoto Y, Ichikawa A (1993). Prostanoid receptors and their biological actions. Prog. Lipid Res..

[19] Ungrin MD (2001). Key structural features of prostaglandin E(2) and prostanoid analogs involved in binding and activation of the human EP(1) prostanoid receptor. Mol. Pharmacol..

[20] Suzawa T (2000). The role of prostaglandin E receptor subtypes (EP1, EP2, EP3, and EP4) in bone resorption: an analysis using specific agonists for the respective EPs. Endocrinology.

[21] Prasanna G (2011). Effect of PF-04217329 a prodrug of a selective prostaglandin EP(2) agonist on intraocular pressure in preclinical models of glaucoma. Exp Eye Res..

[22] Schachar RA, Raber S, Courtney R, Zhang M (2011). A phase 2, randomized, dose-response trial of taprenepag isopropyl (PF-04217329) versus latanoprost 0.005% in open-angle glaucoma and ocular hypertension. Curr. Eye Res..

[23] Kirihara T (2018). Pharmacologic characterization of omidenepag isopropyl, a novel selective EP2 receptor agonist, as an ocular hypotensive agent. Invest. Ophthalmol. Vis. Sci..

[24] Aihara M (2020). Omidenepag isopropyl versus latanoprost in primary open-angle glaucoma and ocular hypertension: the phase 3 AYAME study. Am. J. Ophthalmol..

[25] Taketani Y (2014). Activation of the prostanoid FP receptor inhibits adipogenesis leading to deepening of the upper eyelid sulcus in prostaglandin-associated periorbitopathy. Invest. Ophthalmol. Vis. Sci..

[26] Miller CW, Casimir DA, Ntambi JM (1996). The mechanism of inhibition of 3T3-L1 preadipocyte differentiation by prostaglandin F2alpha. Endocrinology.

[27] Mater MK, Pan D, Bergen WG, Jump DB (1998). Arachidonic acid inhibits lipogenic gene expression in 3T3-L1 adipocytes through a prostanoid pathway. J. Lipid Res..

[28] Casimir DA, Miller CW, Ntambi JM (1996). Preadipocyte differentiation blocked by prostaglandin stimulation of prostanoid FP2 receptor in murine 3T3-L1 cells. Differentiation.

[29] Bonnans C, Chou J, Werb Z (2014). Remodelling the extracellular matrix in development and disease. Nat. Rev. Mol. Cell Biol..

[30] Mouw JK, Ou G, Weaver VM (2014). Extracellular matrix assembly: a multiscale deconstruction. Nat. Rev. Mol. Cell Biol..

[31] Kadler KE, Hill A, Canty-Laird EG (2008). Collagen fibrillogenesis: fibronectin, integrins, and minor collagens as organizers and nucleators. Curr. Opin. Cell Biol..

[32] Okada M, Yamawaki H (2019). A current perspective of canstatin, a fragment of type IV collagen alpha 2 chain. J. Pharmacol. Sci..

[33] Gregorio I, Braghetta P, Bonaldo P, Cescon M (2018). Collagen VI in healthy and diseased nervous system. Dis. Model Mech..

[34] Duggan S (2018). Omidenepag isopropyl ophthalmic solution 0.002%: first global approval. Drugs.

[35] Hollo G, Aung T, Cantor LB, Aihara M (2020). Cystoid macular edema related to cataract surgery and topical prostaglandin analogs: mechanism, diagnosis, and management. Surv. Ophthalmol..

