# Supplementary material for: Omidenepag, a non-prostanoid EP2 receptor agonist, induces enlargement of the 3D organoid of 3T3-L1 cells
Source: Sci Rep. 2020 Sep 29;10:16018. doi: 10.1038/s41598-020-72538-x (PMC7524797; doi:10.1038/s41598-020-72538-x)
Supplement: Supplementary file 1 — Supplementary Information. [file 41598_2020_72538_MOESM1_ESM.pdf]

Supplementary Information

Omidenepag, a non-prostanoid EP2 receptor agonist, induces enlargement of the 3D organoid of 3T3-L1 cells

Key words: deepening of the upper eyelid sulcus (DUES), 3T3-L1cell, omidenepag, omidenepag isopropyl, 3-dimension (3D) tissue culture

Yosuke Ida, Fumihito Hikage, Araya Umetsu, Haruka Ida, Hiroshi Ohguro.

Departments of Ophthalmology, Sapporo Medical University School of Medicine

All correspondence should be addressed to Fumihito Hikage

Tel# 81-11-611-2111, Fax# 81-11-613-6575, e-mail: fuhika@gmail.com

Both authors (Y. I and F. H.) contributed equally to this manuscript.

SUPPLEMENTAL FIGURE 1. mRNA expression of FP (*Ptgfr*) or EP (*Ptger2*) receptor of the 2D and 3D cultured 3T3-L1 cells with adipogenesis.

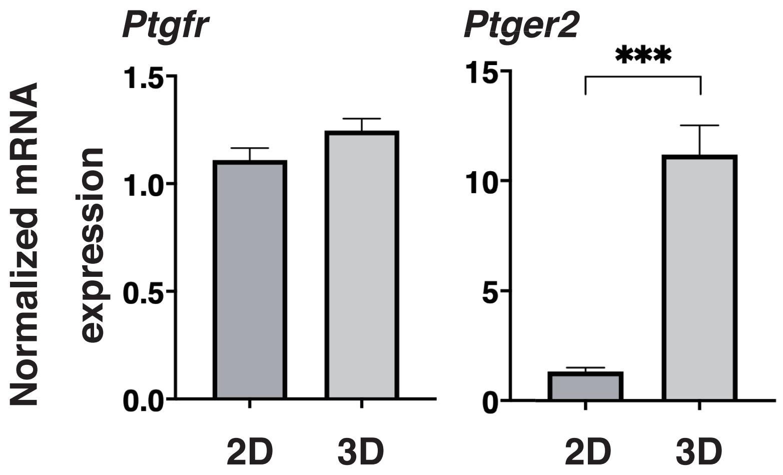

At Day 7, 2D or 3D cultures 3T3-L1 cells with adipogenesis (DIF+) were subjected to qPCR analysis to estimate mRNA expression of FP (*Ptgfr*) or EP (*Ptger2*) receptor. All experiments were performed in duplicate using fresh preparations. Data are presented as the arithmetic mean ± standard error of the mean (SEM). *** P<0.005 (ANOVA followed by a Tukey's multiple comparison test).

Supplementary Table

Gene	Forward primer (5' to 3')	Reverse primer (5' to 3')	TaqMan probe (5' to 3')
**Mouse**			
Pparγ	CTGCTCCACACTATGAAGACAT	TGCAGGTTCTACTTTGATCGC	/FAM/AGCTGACCC/ZEN/AATGGTTGCTGATTACA/IABkFQ/
Cebpα	ACAAGAACAGCAACGAGTACC	TCATTGTCACTGGTCAACTCC	/FAM/CGCAAGAGC/ZEN/CGAGATAAAGCCAAAC/IABkFQ/
Ap2	AAATCACCGCAGACGACAG	CCTTTCATAACACATTCCACCAC	/FAM/ TGAAGAGCA/ZEN/TCATAACCCTAGATGGCG/IABkFQ/
Glut4	TCTTATTGCAGCGCCTGAG	GAGAATACAGCTAGGACCAGTG	/FAM/TGGAAACCC/ZEN/GACGGCATCTTGT/IABkFQ/
Col1a1	CGCAAAGAGTCTACATGTCTAGG	CATTGTGTATGCAGCTGACTTC	/FAM/CCGGAGGTC/ZEN/CACAAAGCTGAACA/IABkFQ/
Col4a1	TCTGGCTGTGGAAAATGTGA	AATCCAATGACACCTTGCAAC	/FAM/TCTTTCTCC/ZEN/CTTTTGTCCCTTCACGC/IABkFQ/
Col6a1	CCAGATGAGTGTGAGATCCTG	AAGTTCTGTAGGCCAATGCTC	/FAM/ACCCATTGA/ZEN/CATCCTCTTCGTGCTG/IABkFQ/
Fn1	GAGCTATCCATTTCACCTTCAGA	TTGTTCGTAGACACTGGAGA	/FAM/CAGGAGATT/ZEN/TGTTAGGACCACGGCA/IABkFQ/
Ptgfr	GCCATAATGTGCGTCTCCT	GATCTGATTCCACFTTFCCA	/FAM/TGGAGTCCC/ZEN/TTTCTGGTAACAATGGC/IABkFQ/
Ptger2	AGAGGAGAGAGGACTTCGATG	GAGGTTTCATCCATGTAGGCA	/FAM/ACCATCACC/ZEN/TTCGCCATATGCTCC/IABkFQ/
36b4	TTATAACCCTGAAGTGCTCGAC	CGCTTGTACCCATTGATGATG	/FAM/AGGCCCTGC/ZEN/ACTCTCGCTT/IABkFQ/

Sequences of primers and Taqman probes used are shown.

Supplementary Movie 1

A single 3D organoid placed on a 3-mm × 3-mm plate was compressed to 50% deformation during 20 sec. The organoids were continuously monitored by a microscopic camera. S: sensor of the mechanical force (μN), P: compression plate, O: single 3D organoid
